# Changes in the Complexity of Limb Movements during the First Year of Life across Different Tasks

**DOI:** 10.3390/e24040552

**Published:** 2022-04-15

**Authors:** Zuzanna Laudańska, David López Pérez, Alicja Radkowska, Karolina Babis, Anna Malinowska-Korczak, Sebastian Wallot, Przemysław Tomalski

**Affiliations:** 1 Institute of Psychology, Polish Academy of Sciences, Jaracza 1, 00-378 Warsaw, Poland; a.radkowska@psych.pan.pl (A.R.); kbabis@psych.pan.pl (K.B.); a.malinowska-korczak@psych.pan.pl (A.M.-K.); 2 Graduate School for Social Research, Polish Academy of Sciences, Nowy Świat 72, 00-330 Warsaw, Poland; 3 Institute of Psychology, Leuphana University of Lüneburg, Universitätsallee 1, 21335 Lüneburg, Germany; sebastian.wallot@leuphana.de

**Keywords:** complexity, motor development, multidimensional recurrence quantification analysis, infants, limb movements

## Abstract

Infants’ limb movements evolve from disorganized to more selectively coordinated during the first year of life as they learn to navigate and interact with an ever-changing environment more efficiently. However, how these coordination patterns change during the first year of life and across different contexts is unknown. Here, we used wearable motion trackers to study the developmental changes in the complexity of limb movements (arms and legs) at 4, 6, 9 and 12 months of age in two different tasks: rhythmic rattle-shaking and free play. We applied Multidimensional Recurrence Quantification Analysis (MdRQA) to capture the nonlinear changes in infants’ limb complexity. We show that the MdRQA parameters (entropy, recurrence rate and mean line) are task-dependent only at 9 and 12 months of age, with higher values in rattle-shaking than free play. Since rattle-shaking elicits more stable and repetitive limb movements than the free exploration of multiple objects, we interpret our data as reflecting an increase in infants’ motor control that allows for stable body positioning and easier execution of limb movements. Infants’ motor system becomes more stable and flexible with age, allowing for flexible adaptation of behaviors to task demands.

## 1. Introduction

One of the fascinating phenomena in human development is how quickly infants learn new motor skills. Infants’ movements advance from being disorganized to having a more recognizable adult-like pattern in the first years of life [1]. The development of motor behavior involves learning through practice as infants improve their skills over time and learn to optimize their actions to the demands of any specific task. However, how motor coordination patterns emerge in development and across different actions is unknown.

Initially, reflexes and general movements are controlled at the spinal and brain stem levels during the neonatal period. Later, motor control at the subcortical level of the central nervous system emerges and matures mainly throughout the first year of life, followed by the activation of the cortical level of motor control [2]. The increase in motor control allows for body positioning and stability, which also facilitates the execution of limb movements [3,4]. Initially, the pattern of spontaneous movements seems to involve all the limbs simultaneously, and it refines to a more selective inter-limb coordination with age [5,6]. The dissociation between arms and legs mainly emerges in the second half of the first year [7], facilitating object manipulation and playing with toys [8]. Moreover, the leg activity becomes more stable with age, while the inverse pattern is observed in the arms [9].

Additionally, the increase in postural control allows for using upper limbs for purposes other than the stabilization of body position. Infants aged 6 and 7 months present trunk control mostly in the thoracic region [10], and the acquisition of trunk control in the lumbar region between 4 and 6 months of age has a positive impact on the quality of reaching behavior [11]. Full trunk control is presented by infants from 8 to 9 months of age [10]. The emerging postural control is also characterized by increasing complexity, where the upper limbs become more involved in skilled manual reaching and less in stabilizing the body posture [12]. During the first three or four months after birth, infants’ head and trunk control are poor, and they mainly lie down if not supported. Around 6 months of age, infants begin to gain sufficient stability to sit independently, allowing them to move their arms more freely. Later, around 8–9 months of age, most infants learn the first ways of locomotion, such as crawling. Finally, towards the end of their first year, infants stand freely and walk around, opening new possibilities to explore the environment.

Motor control development always occurs in a rapidly changing environment consisting of constant constraints (e.g., gravity) and variable and constantly changing elements, such as objects or people. Thus, to understand the development of the complexity of limb movements, we need to consider that they are embedded in a given context and constrained by situational demands [13]. On the one hand, particular contexts may encourage highly structured and repetitive patterns of limb movements—for example, rhythmic activities such as drumming or rattle shaking. Infants’ movements during drumming become faster and more regular with age [14], and the rhythmic synchronization is usually not limited to arm movements but diffuses throughout the body [15]. This increase in the regularity of movements may result in a developmental decrease in the complexity of limb movements. On the other hand, the lack of structure in unconstrained free play may be related to a developmental increase in the complexity of limb movements as older infants can selectively use hands in varied ways to manipulate objects while using legs to stabilize their position or move around. Thus, the context and task demands are also important when evaluating the complexity of limb movements.

The rapid evolution of wearable devices has opened new avenues for recording and analyzing infant movement, which might help to understand the changes in the complexity of infants’ spontaneous movements during different activities in greater detail. Advanced wearable sensors—Inertial Motion Units (IMUs)—combine information from accelerometers, gyroscopes and magnetometers, resulting in a more precise estimation of the position and orientation of body parts. Given the portability, mobility, small size and low weight of this wearable technology, it is becoming widely used in infant studies (e.g., [16,17,18,19,20,21]). Although wearable sensors may cause some discomfort in clinical pediatric populations (as suggested in [22]), studies in typically developing infants have reported that wearables do not affect infant movement (e.g., [23]). An alternative method is using marker-less algorithms to detect movements from videos (e.g., [24,25,26,27,28]). However, this approach is challenging in multi-person set-ups with older infants that move around freely since obtaining a clear view of them at all times remains difficult and the resulting occlusions may significantly limit the accuracy of tracking ([26]). Therefore, the IMUs can currently be considered a gold standard for quantifying infants’ 3D kinematics in multi-person and unconstrained settings.

In this study, we use wearables to investigate the developmental changes in the complexity of limb movements in two tasks that differ in the level of structuring—more constrained rattle-shaking and free play with a larger set of toys. Parent–infant dyads were invited to the lab four times: when infants were around 4, 6, 9 and 12 months age, as these ages reflect significant changes in motor control and gross motor development. As Abney et al. [9] demonstrated, infant development can be studied as a complex system with the analytical tools derived from nonlinear dynamics. Studies on motor development have traditionally focused on quantifying changes in individual limb movements (i.e., reaching hand) or in pairs (either hands or legs). Since the pattern of spontaneous movements initially involves all the limbs shifting simultaneously and it refines with age, in this paper, we focus on the changes in the movement complexity of all limbs together. To achieve this, we use the Multidimensional Recurrence Quantification Analysis (MdRQA, [29]). Many methods of inferring complexity measures from a time series allow for the inclusion of a maximum one (e.g., fractals, recurrence quantification analysis, entropy measures) or two (e.g., cross-recurrence quantification analysis) time series and cannot be used to determine potential higher-level interactions in the movement of all limbs together. MdRQA, in contrast to other methods, is a dynamical systems method that allows for quantifying the dynamics of a multidimensional system at different levels of description by combining information from multiple variables (n > 2) and can be used to infer the shared dynamics of multiple time series [29]. Those shared dynamics are later summarized in a series of parameters that provide information about the complexity of the time series (see description in Section 2.5). Here, we combine wearable motion trackers and MdRQA to study the developmental trajectories of the complexity of infants’ limb movements in two play contexts: rattle-shaking and free play. To our knowledge, the coordination between all four limbs has not been previously studied simultaneously in a longitudinal design and across tasks that differ in their level of constraints. We hypothesize that the trajectories of the complexity of limb movements will differ between the tasks, with the age-related decrease in complexity in the rattles task and the increase in complexity in the free play task.

## 2. Materials and Methods

### 2.1. Participants

Participants were 26 mother–infant dyads from an ongoing longitudinal study. Participants were invited to the lab when infants were around 4 (T1), 6 (T2), 9 (T3) and 12 (T4) months old. Four infants contributed data at all four time points, whereas nineteen infants missed one visit (mostly due to COVID-19 related restrictions). Therefore, 12 infants contributed data at T1, T2 and T3; 7 at T2, T3 and T4; and 3 at T1, T3 and T4 (see Table 1 for an overview of sample characteristics). Participants were from predominantly middle-class families living in a city with >1.5 million inhabitants. The majority of the mothers had completed higher education: 22 held a master’s degree, 2 held a bachelor’s and 2 completed high school. For their participation, infants received a diploma and a small gift (a baby book). The study received clearance from the local institution’s ethics committee.

### 2.2. Equipment

**Figure 1:**
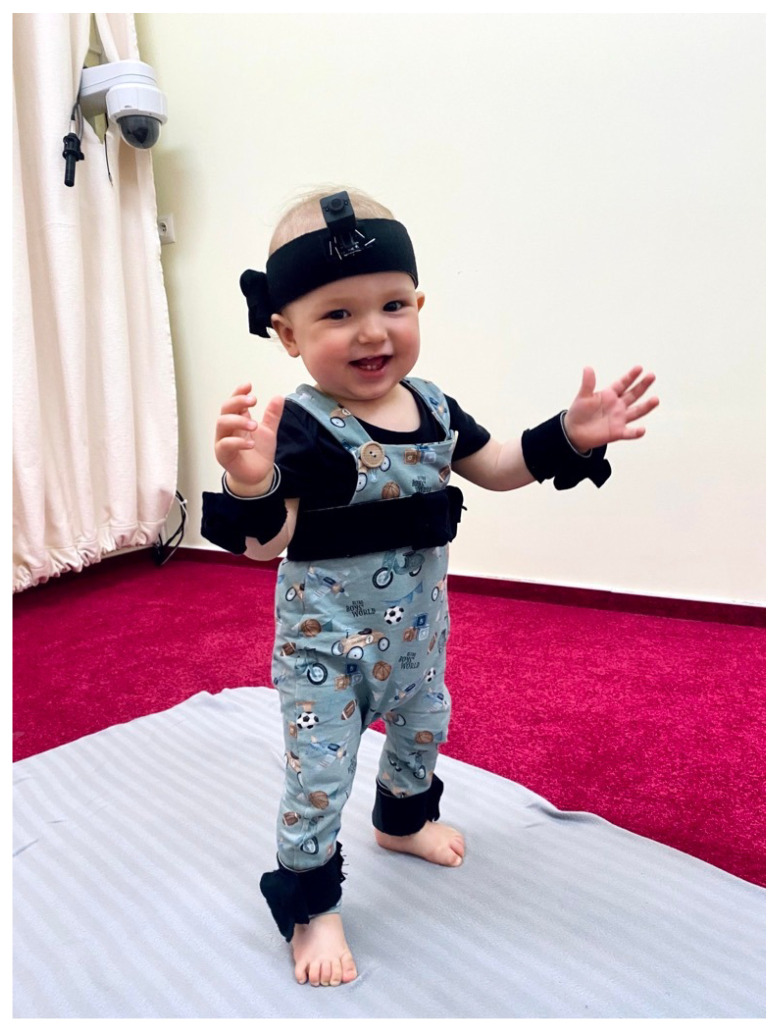
Placement of infant’s motion trackers: legs, hands, torso, head. Signed permission of the caregiver was acquired for the publication of the image.

Infants’ and caregivers’ movements were recorded at 60 Hz using wearable motion trackers (MTw Awinda, Xsens Technologies B.V., Enschede, The Netherlands): an Awinda station receiver (Xsens Technologies B.V.) and MT Manager Software (Xsens Technologies B.V.). Overall, 12 sensors were used (on infant’s arms, legs, head and torso, see Figure 1, and on caregiver’s arms, head and torso), but in this paper, we report data only from 4 sensors placed on infant’s arms and legs.

### 2.3. Procedure

**Figure 2:**
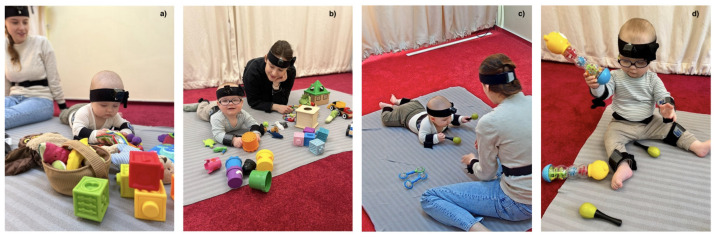
Photos of the toys used in the free play at T1 and T2 (**a**) and T3 and T4 (**b**) and the rattle-shaking task at T1 and T2 (**c**) and T3 and T4 (**d**). Signed permission of the caregiver was acquired for the publication of the images.

Interactions were recorded in a laboratory room on a carpeted play area. Upon the family’s arrival, an experimenter explained the study protocol and obtained parental consent. Once the infant was familiarized with the laboratory, the wearable motion trackers attached to the elastic bands were put on the infant’s and caregiver’s bodies. Then, a set of parent–child interaction tasks with different sets of age-appropriate toys took place. The sets for infants aged 4 and 6 months were slightly different from those for infants aged 9 and 12 months to maintain their interest in a given task (see Figure 2). There were 6–7 different tasks during each meeting, but here, we report data comparing two of them—free play and rattle-shaking. In a rattle-shaking task, which lasted approx. 5 min, the dyads were given two maracas rattles and two rattles of different types (the barbell rattles for younger infants and teddybear rattles for older ones). In a free play task, which lasted approx. 10 min, the younger infants were offered a large, standard set of toys that included baby books, teethers, rattles, rubber blocks and plush toys. The set for older infants included block sorter, cars, stackable cups, rubber blocks, puppets, rattles, plush toys and a wooden box with a drawer and a ball. Caregivers were instructed to play with their infants using each set of toys in their preferred way, as they usually do at home.

### 2.4. Data Pre-Processing

**Figure 3:**
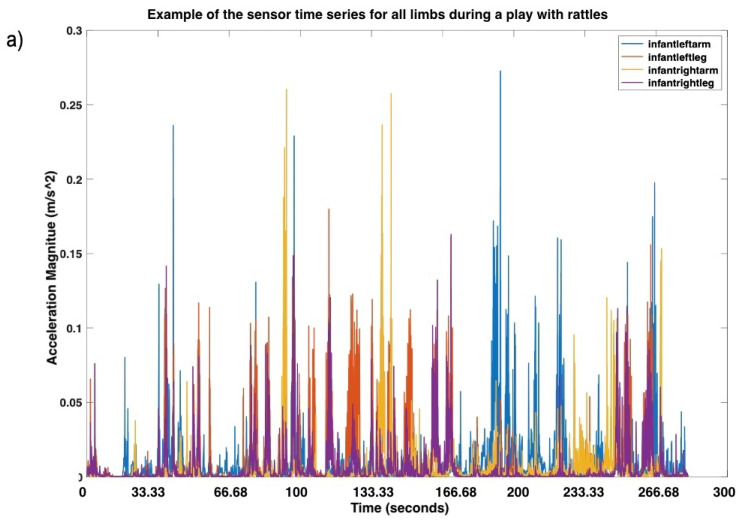

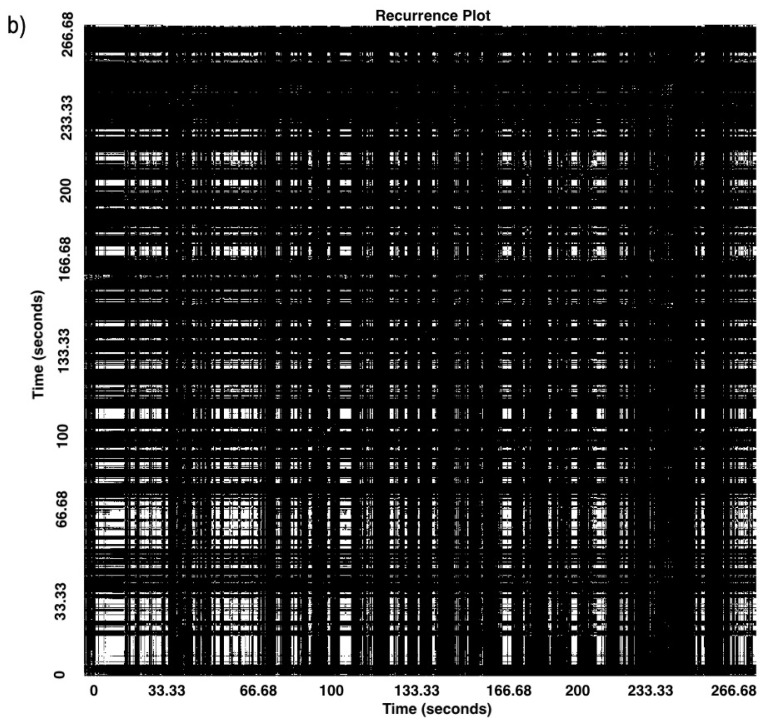
Examples of the sensor time series for all limbs during a play with rattles (**a**) and its correspondent recurrence plot (**b**). Recurrences in the plot are marked by a white dot, while non-recurrences are marked by a black dot.

IMU data from sensors placed on both wrists and ankles of an infant were processed in Matlab (Mathworks, Inc., Natick, MA, USA) using in-house scripts. First, missing values were identified and interpolated using the *interp1* function with a Spline interpolation that applies a cubic interpolation of the values at neighboring grid points. Then, we collapsed the three-dimensional acceleration data obtained from the IMUs to a one-dimensional overall acceleration time series by calculating the magnitude of acceleration for each three-dimensional data point. Next, data were smoothed using the *medfilt1* function that applies a third-order median filter to remove one-point outliers by replacing each value with the median of three neighboring entries (see Figure 3a for an example of the sensor time series). Finally, to avoid the possibility that data from any limb with higher variance may bias the outcome of the complexity analysis and because we were interested in the sequential properties of the data, each individual time series was *z*-scored before further analysis.

IMUs record gyroscopic and magnetometer data, providing more detailed orientation information. Combining this information with accelerometer data, one can create quaternions [30], an alternate way to describe orientation or rotations on limb movements. Supplementary data using quaternions are included to test the robustness of the IMUs data (see Supplementary Information 1.1–1.3).

### 2.5. Complexity Analysis

We used MdRQA [29] to quantify the simultaneous coupling of four limbs’ time series. MdRQA is a multivariate extension of Recurrence Quantification Analysis that captures recurring patterns within a multidimensional time series. This is achieved by calculating the distances between all coordinate pairs of data points (e.g., using Euclidean distance norm) in a multidimensional time series and by thresholding this distance matrix, where distances below the threshold are treated as recurrent, and distances exceeding the threshold are treated as nonrecurrent [31]. That thresholded matrix is called the recurrence plot, where values are coded as 1 or 0 depending on whether the values are recurrent or not for each of the values within the all time series (see Figure 3b for an example). From the final recurrence plot, we extracted three main measures:

- Entropy (Ent): it is the Shannon entropy of the distribution of the diagonal lines on the recurrence plot, capturing repeating movement patterns;
- Recurrence Rate (RR): it is the density of recurrence points in a recurrence plot, and it corresponds to the probability that a specific state will recur;
- Mean Line (ML): it is the average length of repeating patterns in the system, which can be understood as a measure of overall system’s stability.

These three measures allow for describing different yet supplementary aspects of the system’s behavior, such as stability and adaptability. When infants acquire a new motor skill, their repertoire becomes more complex allowing for increased adaptability to situational demands (e.g., [32]). Furthermore, when infants master these new skills, their motor coordination patterns become more stable over time. In this context, entropy acts as a measure of the complexity or flexibility of limb movements. An unconstrained movement signal will carry low entropy since the probability of finding recurrent patterns would be lower than in a constrained situation with interaction-dominant dynamics, which postulates that the system’s structure is emergent and context-dependent. In contrast, component-dominant dynamics proposes that all components (in this case, infants’ limbs) contribute to the system dynamics in a stable and independent way [33,34,35]. When infants are playing in an unconstrained situation (free play task in our study), they adjust their movements to the needs of the task at hand, i.e., perform various types of movement (e.g., reaching, banging, touching) with different types of objects. Consequently, their movements are less regular and form more random patterns. In this case, there will be low variability in the length of the recurrent states, leading to lower entropy. In the constrained situation (rattle-shaking task), infants move their arms in a rhythmic way to produce the sound, and the rattles placed in their hands may reduce the number of degrees of freedom of movement. Therefore, the rattle-shaking task decreases in complexity as infants attempt to perform periodic/semi-periodic movements, introducing higher variability in the patterns of recurrences and increasing the overall entropy. On the other hand, the recurrence rate and mean line are measures of the stability of the limb movements. In a constrained situation, such as rattle-shaking, the more the infants’ movements would follow interaction-dominant dynamics (i.e., the infants learn with age how to move the rattles synchronously), the more recurrence rate and mean line would increase.

Three critical parameters need to be set to calculate the recurrence plots (see [36]). First, we estimated the delay of embedding using the *mdDelay* function, which estimates the delay in a multidimensional time series using the average mutual information method. Second, we estimated the embedding dimension using the *mdFnn* function, which applies a false nearest neighbor estimation. We obtained an average value of 1 for the delay and 14 for the embedding dimension, which is consistent with the typical values recommended for biological signals [37]. Finally, we adapted the radius for each infant individually. To this end, we fixed the recurrence rate sufficiently low (i.e., RR = 5% [38] and used the embedding dimension and delay previously computed. We carried this out for the first visit data of each infant and fixed these parameters for the consequent visits to estimate the changes in complexity over time.

Control analyses were performed using the same approach but with shuffling the movement data in a random order within each time series. This allows us to compare the results from the entropy and mean line and prove that temporal dynamics did not arise randomly (e.g., [39]).

### 2.6. Statistical Analysis

**Figure 4:**
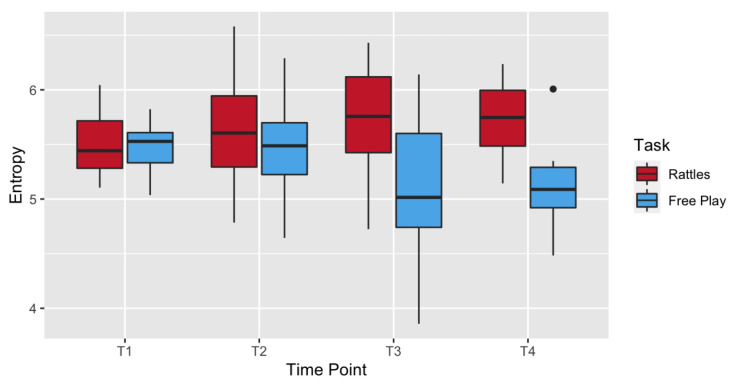
Boxplots showing entropy in each time point in rattle-shaking (red) and free play (blue). Horizontal lines represent median value, boxes are drawn from the first quartile to the third quartile, whiskers indicate min and max value and the dot indicates an outlier.

To assess the repeated-measures effects of age (4) and task (2), we ran the General Estimating Equations (GEEs) with a Bonferroni correction for pairwise comparisons. GEEs are particularly adequate for longitudinal data because they take into account the dependency and ordering of the data within subjects in repeated-measures designs. Furthermore, in the GEE analysis, even if a subject is missing one or more of the repeated measurements, the remaining data of that subject are used in the analysis (e.g., [40,41]). Data analysis was conducted in IBM SPSS Statistics 26. Figure 4 was created using R [42] and RStudio [43] and ggplot2 package [44].

## 3. Results

### 3.1. Complexity Measures

#### 3.1.1. Entropy

The GEE with age (4) and task (2) as within-subjects factors showed a significant difference in entropy level between rattle-shaking and free play (Wald $\chi^{2}$(1) = 36.888, *p* < 0.001; see Figure 4). There was no effect of time point (Wald $\chi^{2}$ (3) = 3.365, *p* = 0.339), but the interaction between task and time point was significant (Wald $\chi^{2}$ (3) = 26.634, *p* < 0.001). Post hoc pairwise comparisons revealed that there were no task-related differences at T1 and T2. The entropy was higher in rattle-shaking than free play at T3 (*p* < 0.001) and T4 (*p* = 0.010). Within free play, entropy was also higher at T2 than at T3 (*p* = 0.037). See Table 2 for descriptive data.

#### 3.1.2. Recurrence Rate

There was a significant difference in the recurrence rate between rattle-shaking and free play (Wald $\chi^{2}$ (1) = 11.281, *p* = 0.001). There was no effect of time-point (Wald $\chi^{2}$ (3) = 4.353, *p* = 0.226), but the interaction between task and time-point was significant (Wald $\chi^{2}$ (3) =18.660, *p* < 0.001) as the recurrence rate in rattle-shaking at T3 was significantly higher than in free play at T3 (*p* = 0.001) and T4 (*p* = 0.029).

#### 3.1.3. Mean Line

There was a significant difference in the mean line between rattle-shaking and free play (Wald $\chi^{2}$ (1) = 8.919, *p* = 0.003). The interaction effect between task and time-point was also significant (Wald $\chi^{2}$ (3) =17.739, *p* < 0.001) as the mean line in free play at T3 was lower than in rattle-shaking at T3 (*p* < 0.001). There was no effect of time-point (Wald $\chi^{2}$ (3) = 3.618, *p* < 0.306).

### 3.2. Control Analysis

To check whether the effects did not arise randomly, we compared observed and shuffled versions using paired t-tests at each time point. At each time point, the observed versions were significantly different from those shuffled for each measure. At T1: entropy t(34) = 76.675, *p* < 0.001; recurrence rate t(34) = 162.090, *p* < 0.001; mean line t(34) = 21.334, *p* < 0.001. At T2: entropy t(45) = 79.094, *p* < 0.001; recurrence rate t(45) = 9.841, *p* < 0.001; mean line: t(45) = 19.053, *p* < 0.001. At T3: entropy: t(48) = 53.352, *p* < 0.001; recurrence rate t(48) = 7.580, *p* < 0.001; mean line t(48) =16.382, *p* < 0.001. At T4: entropy t(22) = 43.767, *p* < 0.001; recurrence rate t(22) = 5.331, *p* < 0.001; mean line t(22) =14.276, *p* < 0.001.

## 4. Discussion

In this paper, we showed that the complexity of limb movements changes across infancy. In a longitudinal study, we recorded infants’ limb movements at around 4, 6, 9 and 12 months of age in two tasks that differed structurally—more constrained and repetitive rattle-shaking and free play with a larger set of toys. To investigate the changes in the complexity of all four limbs, we applied the Multidimensional Recurrence Quantification Analysis (MdRQA, [29]). We showed that the complexity measures (entropy, recurrence rate and mean line) are modulated by task at 9 and 12 months but not at 4 or 6 months of age. We interpret this finding as reflecting an increase in infants’ motor control that allows for stable body positioning and easier execution of limb movements. Increased motor control is related to an overall increase in the motor system’s complexity as the infant can adjust movements specifically to the task. In our case, higher entropy in the rattle-shaking task may reflect the capacity to flexibly adapt behaviors to environmental demands. Furthermore, a longer mean line and a higher recurrence rate suggest that an infant’s motor system is more stable during rattle-shaking and has a more confined attractor state.

Our results provide further insight into the early developmental organization of motor actions. The global pattern of inter-limb coordination varies with changing contexts because the behaviors are adapted and selected to fit a given task [1]. The motor action system continues to specialize across infancy to respond to particular environmental pressures [45]. In our case, each task qualitatively required different acts—rhythmic body movements to produce the rattling sound or various reaching and holding acts to explore different objects—and infants learned how to adjust their behaviors to the specific context with age. This suggests that limb movement organization becomes context-specific by the end of the first year of life. This is in line with recent studies showing that less experienced infants generate multiple inconsistent coordination patterns, while more experienced infants tailor their coordination patterns to body–environment relations and flexibly switch solutions (e.g., [32,46,47]).

This study is an important step in understanding changes in the complexity of limb movements in infancy. We showed that the MdRQA measures are sensitive to changes in the dynamics of limb movements between tasks and that the observed patterns do not form randomly, as was shown in comparisons with the shuffled time series. This result is in line with previous studies suggesting that infants’ development can be studied as a complex system with the tools from nonlinear dynamics [9]. Moreover, MdRQA goes one step further than traditional methods as it allows estimating the complex dynamics of multiple effectors (n > 2) and, therefore, characterizing the complexity of the developmental organization of motor actions in more detail. Nevertheless, MdRQA can be further extended to assess the coupling between multidimensional time series [48]. Therefore, methods such as MdRQA open new possibilities to understand the role of limb movement for other domains of development (e.g., vocal production or visual attention) or studying coupling and leader–follower relationships during parent–infant interactions (e.g., parent limb movements vs. infant limb movements or parent vocalizations vs. infant limb movements).

Several limitations arise from this study. First, there were some missing values in the sensor data in 10.1% of cases. However, control analysis with excluded cases with over 15% of missing data showed the same final pattern of the results (see Supplementary Information 2.1–2.3). Second, we used only accelerometer data in this study, while IMUs offer more possibilities (magnetometer and gyroscope data). To establish whether our results are limited to accelerometric data only, we conducted a supplementary analysis using quaternion data and showed a similar pattern of results with respect to task modulation and age-related changes (see Supplementary Information 1.1–1.3), but further studies should consider the possibility of expanding this work and explore not only changes in acceleration but also rotational movements. Third, in this study, we compared tasks that differed in overall duration (5 min in rattle-shaking vs. 10 min in free play). Variable length of analyzed time series are commonly used in studies using RQA-based approaches (see, for example, [24,49]) since capturing the overall dynamics of the phenomenon is more important than task duration (and comparison of observed data with shuffled time series allows checking whether the effects were not random). Fourth, although we observed different age-related trajectories in the complexity of limb movements over the first year of life, there is high variability in the way infants develop. Therefore, future longitudinal studies with more time-points are necessary to more accurately depict the patterns of variability and the shape of the developmental trajectories of inter-limb coordination. This is especially important since stable execution of gross motor skills is usually preceded by many transitions when the skills vacillate between occurrence and absence [50], which could reflect phase transition periods when the entire motor system undergoes reorganization. Thus, nonlinear methods combined with a more dense sampling of behavior across development could shed more light on the developmental trajectories of movement coordination and capture both phase transitions and periods of stability. Fifth, data were collected in a laboratory room, and therefore, future studies could explore the possibilities of continuous measurement of limb coordination across different contexts “in the wild”. The wearable motion trackers can be worn for the entire day or multiple days without the presence of an experimenter and record densely sampled data during infants’ everyday experiences [17,21]. A dense sampling of infants’ daily experiences would help understand how caregivers scaffold infants’ actions and create “social affordances” [51] and understand the influence of social influences in context-dependent changes in infants’ inter-limb coordination. Moreover, it could also help to identify atypical patterns of motor development. Lower complexity of movements might represent more repetitive motor behaviors, which are diagnostic symptoms of several neurodevelopmental disorders, such as autism spectrum disorder [52] or developmental delay [53]. Finally, future studies should investigate whether a similar pattern of results could be observed using other ways of movement tracking, such as marker-less video-based algorithms (see [24,25,26,27,28]), to make sure that wearing sensors does not affect infant movement.

## 5. Conclusions

Our study explored the developmental changes in the complexity of limb movements in infancy using a multidimensional nonlinear approach (MdRQA). We showed that infants’ movements become more complex with age and that the age-related changes in complexity are context-dependent. We interpret these changes in the complexity of the motor system as an increase in motor control that allows the infant to adjust movements specifically to the task. These findings may have important implications for the study of atypical patterns of motor development.

## Figures and Tables

**Table 1:** Sample Characteristics.

Time Point	N	Mean Age in Months (SD)	Min Age in Months	Max Age in Months
T1	19	4.41 (0.30)	4.00	5.20
T2	21	6.57 (0.36)	6.00	7.20
T3	26	9.14 (0.41)	8.60	10.20
T3	17	12.14 (0.46)	11.60	13.10

**Table 2:** Entropy (Ent), Recurrence Rate (RR) and Mean Line (ML) values at each time point and each task.

		T1			T2			T3			T4		
		Mean (SD)	Min	Max	Mean (SD)	Min	Max	Mean (SD)	Min	Max	Mean (SD)	Min	Max
**Rattles**	***Ent***	5.51 (0.30)	5.10	6.04	5.62 (0.44)	4.78	6.58	5.72 (0.45)	4.72	6.43	5.73 (0.37)	5.14	6.24
	***RR***	5.03 (0.05)	4.93	5.09	7.28 (5.13)	2.07	19.14	9.17 (7.19)	0.69	27.55	7.78 (5.01)	0.95	15.60
	***ML***	19.48 (6.61)	5.07	35.20	23.06 (8.59)	11.96	50.95	23.55 (9.02)	1.66	41.79	23.79 (5.78)	15.13	32.19
**Free Play**	***Ent***	5.46 (0.20)	5.04	5.82	5.48 (0.42)	4.64	6.29	5.10 (0.59)	3.86	6.14	5.08 (0.42)	4.48	6.01
	***RR***	5.05 (0.04)	4.98	5.09	5.47 (3.84)	0.14	14.54	4.51 (4.79)	0.02	16.26	2.99 (3.52)	0.17	13.28
	***ML***	18.96 (2.75)	14.17	23.79	21.04 (7.36)	10.96	39.05	16.15 (6.28)	7.44	30.03	15.39 (4.53)	9.92	26.78

## Data Availability

The data that support the findings will be available upon request from the corresponding authors following an embargo from the date of publication to allow for finalization of the ongoing longitudinal project. The computer code used in this study is openly available in GitHub: https://github.com/Mirandeitor/entropyPaper, accessed on 10 February 2022.

## Supplementary Materials

The following are available online at https://www.mdpi.com/article/10.3390/e24040552/s1.
