# Supplementary material for: Changes in the Complexity of Limb Movements during the First Year of Life across Different Tasks
Source: Entropy (Basel). 2022 Apr 15;24(4):552. doi: 10.3390/e24040552 (PMC9028366; doi:10.3390/e24040552)
Supplement: Supplementary file 1 [file entropy-24-00552-s001.zip › entropy-1611412-supplementary.pdf]

Supplementary Information

Changes in the complexity of limbs movements during the first year of life across different tasks

1. Complexity measures based on quaternions

IMU data offer the possibility of computing quaternions which are mathematical vectors used to rotate and scale an original vector to estimate how much change from one point in space to another has happened. Quaternions include changes in acceleration combined with changes in orientation/rotation and do not suffer from gimbal lock or drifts as some classic accelerometers do. Thus, to establish whether our results are limited to accelerometric data only, we repeated the same analysis with quaternions (the computer code for these calculations is openly available in GitHub: https://github.com/Mirandeitor/entropyPaper).

1.1. Entropy

The GEE with age (4) and play type (2) as within-subjects factors showed a significant difference in entropy level between rattle-shaking and free play (Wald $\chi 2$ (1) = 35.631, $p < .001$). Overall, entropy was higher in rattle task than in free play. There was no effect of time point (Wald $\chi 2$ (3) = 3.914, $p = .271$), but the interaction between task and time point was significant (Wald $\chi 2$ (3) = 26.197, $p < .001$).

1.2. Recurrence Rate

There was a main effect of time-point (Wald $\chi 2$ (3) = 8.732, $p = .033$). There was no significant difference in recurrence rate between rattle-shaking and free play (Wald $\chi 2$ (1) = 3.331, $p = .068$) and the interaction between task and time-point was also not significant (Wald $\chi 2$ (3) = 4.637, $p = .200$).

1.3. Mean Line

There was a significant difference in mean line between rattle-shaking and free play (Wald $\chi 2$ (1) = 38.503, $p < .001$). The interaction effect between task and time-point was also significant (Wald $\chi 2$ (3) =24.292, $p < .001$).There was no effect of time point (Wald $\chi 2$ (3) = 5.196, $p = .158$)

For both entropy and mean line the main and interactions effects were the same as in acceleration-based analysis. However, for recurrence rate we observed a different patterns for quaternions than for acceleration-based analysis. Overall pattern of results is similar, yet there was far more variability in the values observed for quaternions-based recurrence rate (Fig.S1) at T3 and T4 than for acceleration-based (Fig.S2) recurrence rate.

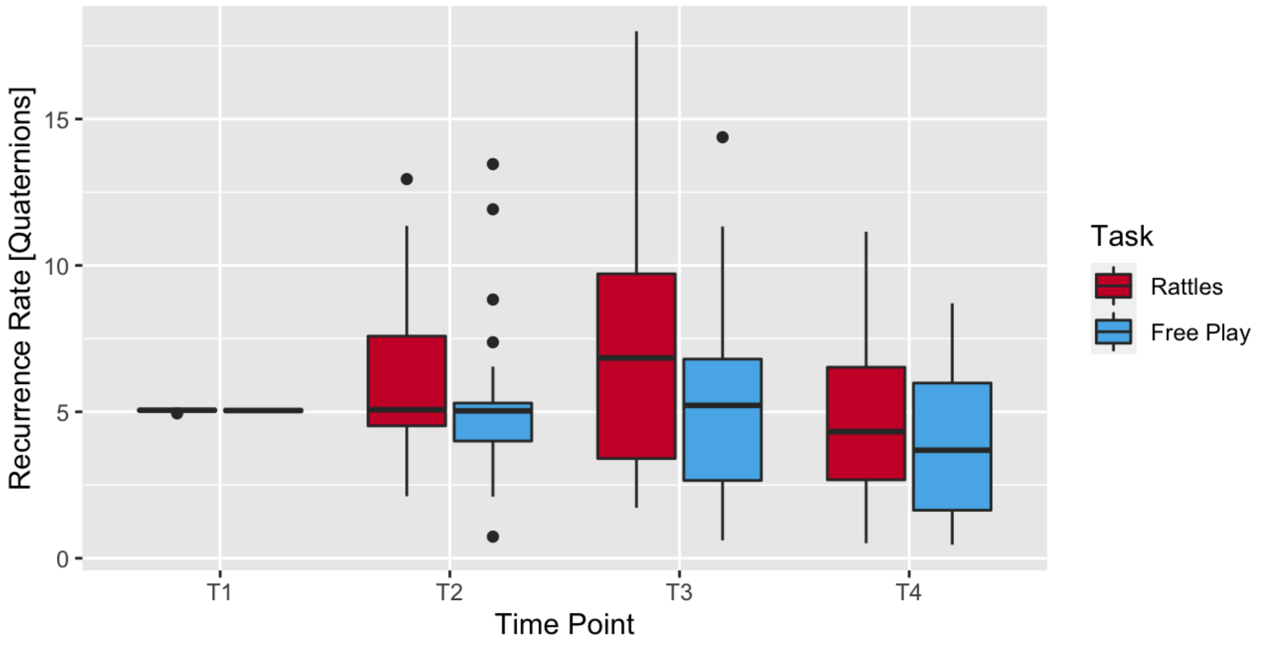

Fig. S1 Boxplots showing recurrence rate in quaternions-based analysis in each time point in rattle-shaking (red) and free play (blue).

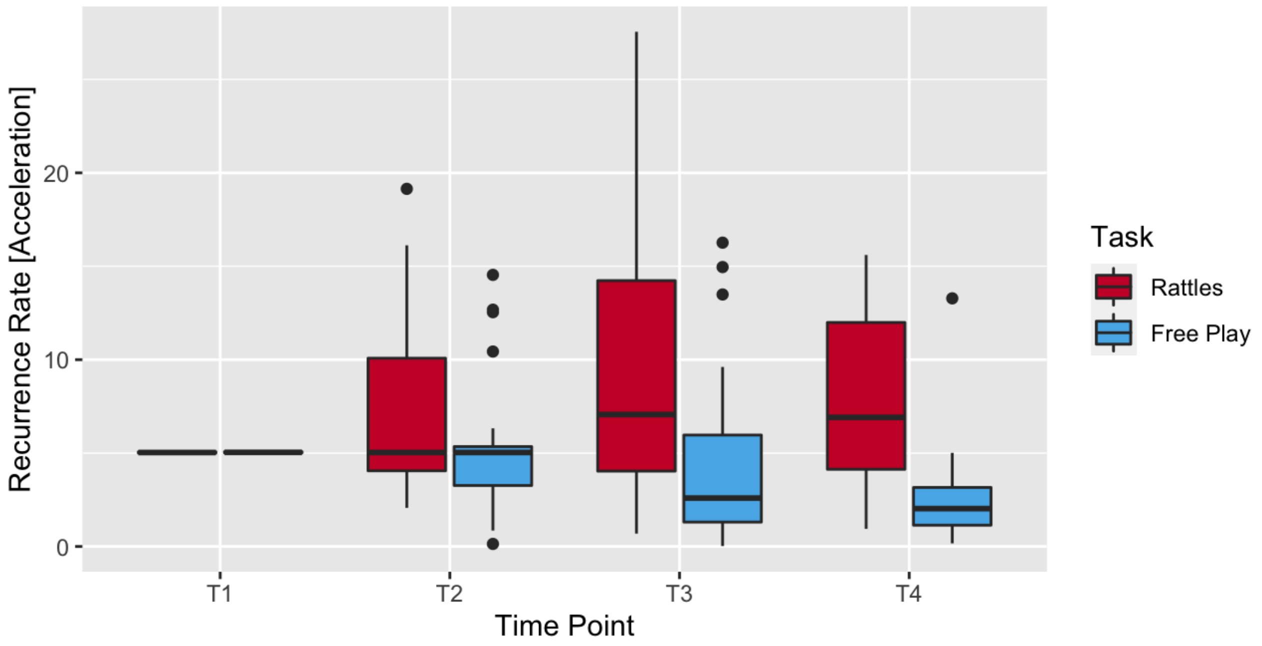

Fig. S2 Boxplots showing recurrence rate in acceleration-based analysis in each time point in rattle-shaking (red) and free play (blue).

2. Complexity measures based on acceleration data - excluded participants with significant amount of missing values in sensor data

In these analyses, we excluded visits in which there was >15% missing sensors data (which consisted of 10.1% of total cases). The significance of all main and interaction effects reported in the paper (section 3.1.) remained unchanged.

2.1 Entropy

The GEE with age (4) and play type (2) as within-subjects factors showed a significant difference in entropy level between rattle-shaking and free play (Wald $\chi2$ (1) = 50.304, $p < .001$; see Fig.3). Overall, entropy was higher in rattle task than in free play. There was no effect of time point (Wald $\chi2$ (3) = 2.534, $p = .469$), but the interaction between task and time point was significant (Wald $\chi2$ (3) = 16.137, $p < .001$).

2.2. Recurrence Rate

There was a significant difference in recurrence rate between rattle-shaking and free play (Wald $\chi2$ (1) = 11.281, $p = .001$). Overall, it was higher in rattle-shaking task than free play. There was no effect of time-point (Wald $\chi2$ (3) = 4.353, $p = .226$), but the interaction between task and time-point was significant (Wald $\chi2$ (3) =18.660, $p < .001$)

2.3. Mean Line

There was a significant difference in mean line length between rattle-shaking and free play (Wald $\chi2$ (1) = 22.616, $p < .001$). There was no effect of time point (Wald $\chi2$ (3) = 1.870, $p = .600$), but the interaction between task and time point was significant (Wald $\chi2$ (3) = 17.659, $p < .001$).
